# Escaping the past and living in the present: a qualitative exploration of substance use among Syrian male refugees in Germany

**DOI:** 10.1186/s13031-021-00352-x

**Published:** 2021-04-12

**Authors:** Jutta Lindert, Ulrike Neuendorf, Marta Natan, Ingo Schäfer

**Affiliations:** 1 Center for Interdisciplinary Addiction Research (ZIS), University of Hamburg, Hamburg, Germany; 2 Department for Psychiatry and Psychotherapy, University Medical Center Hamburg-Eppendorf, Hamburg, Germany; 3 University Hospital Hamburg / Eppendorf, Hamburg, Germany

**Keywords:** Trauma, Mental health, Qualitative research, Refugee populations, Substance use, Escape, Syria

## Abstract

**Background:**

Syrians have been the largest group of refugees in Germany since 2014. Little is known about Syrian refugees` perspectives on substance use. The aim of this study is to investigate the perspective of male refugees from Syria and to foster specific knowledge and understanding of substance use.

**Methods:**

We applied a qualitative study design. Five semi-structured focus group discussions with a total of 19 refugees were conducted in 2019 among the difficult to reach population of Syrian refugees. Audio recordings were translated and transcribed. We used a hybrid approach by integrating inductive and deductive thematic frameworks.

**Results:**

We identified common themes. Firstly, refugees perceived that substances are widely available and accepted in Germany. Secondly, refugees perceived that rules and norms in Germany differ from rules and norms in the home country and favor availability of substances. Thirdly, substance use is related to the intention to escape the past. Fourthly, substance use is related to living in the present through connecting with others and being part of the community. Finally, mental health professional treatment for substance use is associated with shame.

**Conclusions:**

Findings support Syrian refugees` perspectives of substance use as a way of both escaping the past and coping with psychosocial difficulties in the present in a socio-ecological understanding. Understanding the explanatory model of Syrian refugees can inform future interventions to prevent substance abuse and design tailored interventions. Further studies with Syrian refugees in more countries are needed to better understand resettled refugees` perspectives on substance use.

**Supplementary Information:**

The online version contains supplementary material available at 10.1186/s13031-021-00352-x.

## Highlights

- Refugees from Syria perceive that substances in Germany are easily available.
- Perceptions prevail that family norms and societal norms contribute to substance abuse.
- Intentions to escape the past and live in the present are associated with substance use.
- Knowledge of refugees’ perceptions of substance use are necessary to better develop targeted prevention and interventions.

## Introduction

Understanding substance use among forcibly displaced persons remains limited despite the increasing number of forcibly displaced persons globally [1–3] and increasing knowledge on mental disorders among refugees [4–9]. Worldwide, in 2019 around 65 million people have been forcibly displaced because of wars, conflicts and persecution or human rights violations [10]. The high level of forcibly displaced people has mainly been due to conflicts in Venezuela, Afghanistan, South Sudan and the Middle East, in particular in Syria [10]. Over half of the Syrian population has been forcibly displaced since the start of the Syrian conflict in March 2011, with 6 million internally displaced persons (IDPs), 5 million Syrian refugees living in neighboring countries and nearly 1 million seeking asylum in Europe [10]. While the neighboring countries have hosted the majority of the refugees from Syria, around 770,000 have sought refuge in Germany [10]. Refugees from Syria have faced war-related violence, including massacres, murder, torture and stressful situations including poverty and limited access to food in their home country [11]. Furthermore, they often face difficult situations such as poverty, loss of family and community support and lack of employment in displacement [12]. These pre-, during-, and post-flight events have been associated with mental health problems among refugees including problematic substance use [13, 14]. Problematic substance use imposes a significant health burden globally accounting for at least 6.5% of total disability –adjusted life-years and at least five million deaths globally in 2010 [15, 16]. Additionally, problematic substance use may have long-term consequences in functioning and health-related quality of life [16–19] and strategies to reduce the problematic use of substances are needed.

There is limited data on the use of substances (e.g. alcohol, cannabis) in refugees [20–22] Systematic reviews in 2020, 2016 and 2012 found heterogeneous prevalence rates of substance use among refugees [1, 2, 23]. Prevalence rates vary between 11.8% among Bosnian [24] and 15% among Iraqi refugees [25] to 38% among Cambodian refugees [26, 27]. In camp settings, substance abuse prevalence rates vary between 17 and 66% [2, 3]. The prevalence of substance use depends on the context (e.g. availability of substances), the refugee group under study and measures of substance use [16]. Furthermore, substance use habits may differ between groups and change over time in the host countries. For example, alcohol use has been traditionally low in Syria [28–30], while in Europe and specifically in Germany, the consumption of alcoholic beverages is not only seen as socially acceptable, but is relatively accepted as a common habit [31, 32]. However, in many Arab countries, including Syria and in countries that had previously banned the use of alcohol, a shift in social norms has happened in the past five years and alcohol use has become increasingly acknowledged [33]. To understand substance use in refugees, knowledge of explanatory models is critical. Explanatory models refer to ways “that cultural groups experience, understand and communicate suffering, behavioral problems, or troubling thoughts or emotions”. How they view causes and potential outcomes of their problems, including what they believe is appropriate treatment [34].

Explanatory models of substance use differ and include, among others, trauma-focused and psychosocial models. The trauma-focused explanatory model understands substance use as related to traumatic experiences [1, 23]. This approach is in line with research suggesting that substance use disorders are largely comorbid with trauma-related disorders due to pre- and duri*ng* flight life-threatening travel conditions [2, 35]. The refugee experience includes flight from the country of origin, life in a refugee camp, and returning home or third-country resettlement which includes exposure to a range of potentially traumatic events, such as violence and discrimination in the country of origin and in the host country, that negatively influence psychosocial functioning [36, 37]. Research on refugees has identified an association between stressful experiences and substance use. (28, 29, 30, 32, and 31) The trauma-focused approach, however, has limitations as it does not capture the wider consequences of displacement, namely social suffering such as economic insecurity, difficulties to be accepted by the host country and difficulties to adapt in the host country [38, 39].

Social suffering is defined as the amount of human problems that result from what political, economic, and institutional power does to people including human responses to problems as they are influenced by those forms of power [40]. As such social suffering is the product of the context refugees live in such as the cultural, social, political, and event related context [41]. This context includes post-flight experiences such as disruption of protective community and family networks, an unfamiliar environment in the host country, and the need to acquire a new language and find a new professional position [2]. The ecological model stands in the tradition of psychosocial models [42], in which the influence of factors at multiple levels (individual, family, community and society) on mental health is considered [43]. Within that model, substance use of refugees is understood as stemming not only from stressful pre-and during-flight experiences but also from social and material conditions of everyday life following displacement.

Results of epidemiological studies on substance use among conflict affected Syrians need to be interpreted with caution [1]. Usually, available assessment instruments such as the Alcohol Use Disorders Identification Test (AUDIT) [44]; the CAGE questionnaire [45]; the Mini International Neuropsychiatric Interview (MINI) [46]; and the Composite International Diagnostic Interview (CIDI) [47] do not assess local idioms of distress. Investigating explanatory models of substance use, therefore, is an important attempt to acknowledge that perceptions of substance use are contextually shaped [48]. Understanding explanatory models will allow better communication and can be used in designing interventions [49] For the purposes of this paper, we define culture as ‘a system of shared meanings, institutions, and practices’ [50].

To provide better services to refugees the United Nations High Commissioner for Refugees (UNHCR) advised consulting the target groups and investigate their perspectives on mental health as well as ways of coping with past and the present [51]. An investigation into the perspectives qualitative methods was recommended. Following the UNHCR recommendation, this study aims at investigating male refugees’ perspectives on substance use and at fostering knowledge about the specific understanding substance use among refugees from Syria.

## Methods

### Design

This qualitative study is part of a large five-year intervention study (PREPARE), which aims at investigating interventions for refugees using substances in Germany. For this particular study a qualitative design with semi-structured focus group discussions was chosen. Focus groups provide not only an ideal form for communities to share collective opinions [52] but can yield more insights than an equivalent number of individual interviews. Compared to techniques such as individual interviews and surveys, focus groups offer an opportunity to explore topics in context, depth and detail, without imposing a conceptual framework. However, focus groups participants are sometimes reluctant to deal with sensitive topics in a discussion setting compared with an individual interview or survey. Following established recommendations for conducting focus groups, we organized five focus groups with 3–9 participants. We followed the guidelines for reporting qualitative studies [53] (Supplemental material 2).

### Recruitment and participants

A comprehensive recruitment strategy was used by combining purposive and snowball sampling approaches. Local organizations (e.g., psychosocial centers, hospitals, language courses) in a rural area, an urban area and a metropolitan area in Northern Germany, which work with refugees, were contacted. Organizations were informed about the aims of the study and asked to help in recruiting participants. Additionally, local refugees and key-persons (e.g. social workers, deputies for integration and migration) working with refugees were identified. These key persons were asked to help recruit potential participants. Potential participants were then contacted by phone or email and screened as to whether they fulfilled the inclusion criteria. Criteria for inclusion were being over the age of 18, being male, and having arrived to Germany within the last 5 years, i.e. because of seeking refuge from the Syrian war. If they met all the inclusion criteria, they were invited to participate in the study. Additionally, a snowball sampling approach was conducted whereby participants were asked if they knew of any other refugees from Syria and if they could put us into contact with these refugees for initial screening for the inclusion criteria and potential recruitment. Five focus group discussions (FGDs), which included 19 participants, were conducted in three areas in Germany (one metropolitan, one urban, one rural).

### Procedure

We conducted the FGDs between July 2019 and February 2020. Prior to the FGDs, all participants were provided with an invitation leaflet, detailing the discussion topics and research aims of the project. After an invitation by phone or email, the participants had the opportunity to choose a suitable date for the FDGs. A semi-structured interview guide was developed in German. The interview guide was developed to reflect the research aims and concerns reported by refugees. (English translation in Supplemental Material 1).

The FGDs were on average 60–90 min in duration and were conducted with a native-speaking professional Syrian translator, one facilitator (UN) and two observers (student research assistants) in meeting rooms of universities or NGOs working with refugees. The facilitator (UN) guided the group, and the two student research assistants observed and took notes. The languages spoken were German and Arabic. Participants generally spoke German if they felt confident in expressing themselves in the language. The translator assisted on occasions when language barriers occurred or whenever specific terminology was required. With the consent of study participants 1) notes were taken on the printed interview guide to assess assumptions and record important keywords and 2) FDGs were tape-recorded and later transcribed verbatim using Easytranscript software. No individual person could be identified in the audio-recordings; names and places were removed.

### Analyses

FDGs were anonymized, transcribed, and coded using the computer program MaxQDA (version 18) [54], and evaluated using a thematic analysis [55, 56] according to Braun & Clarke [57]. To analyze the data we (UN, JL) used an iterative approach, reviewing interview transcripts of the three rounds of FDGs to draw initial observations related to the research aims. Then, by combining deductive and inductive approaches, an initial set of conceptual codes were identified and systematically applied to blocks of text (usually of three to six sentences in length) [58]. Codes were populated with data in the form of direct quotes from transcripts, and then grouped into key sub-themes and themes. To ascertain whether a code was appropriately assigned, we compared text segments to those which had been assigned the same code previously. Using constant comparisons, we refined dimensions of existing codes and identified new codes. Specifically, the coded data were categorized and linked by relationship into categories. Thereupon, links were established between the categories and defined properties. Names and definitions were developed for each theme and extracts of data most illustrative of the themes were selected for display in the present manuscript. No major new ideas emerged during analysis of the final transcripts, suggesting that data saturation was achieved. For the purpose of this paper, we present the main themes that stood out in FDGs in the results section.

### Research ethics

Ethical clearances for this study were received through the IRB of the University of Applied Sciences Emden/Leer. All necessary steps were taken to ensure participants were thoroughly informed about the project, as well as the protection of the data they provided. The study was conducted in accordance with the principles of the Helsinki Declaration [59] and the EU guidance note “Research on refugees, asylum seekers and migrants” [60]. Participants were included in the study if they gave permission through a consent process, which provided information on confidentiality, data storage, and audio recording. Participants were reminded of their voluntary participation and sharing of in-formation as well as their right to terminate the interview at any point.

## Results

Only male refugees (*n* = 19) participated in the FDGs. Participants were between 20 and 50 years old; due to concerns around anonymity in the rural settings we are not able to provide descriptive statistics for age. No one lived in a refugee camp. We did not assess further socio-demographic data to guarantee confidentiality. No major differences were identified between the five focus groups. Hence, results are commonly reported for all groups. There were three overarching themes with six sub-themes related to understanding perceived causes, risk and protective factors for substance use in the Syrian refugee communities.

### Theme 1: use of substances

**Fig. 1:**
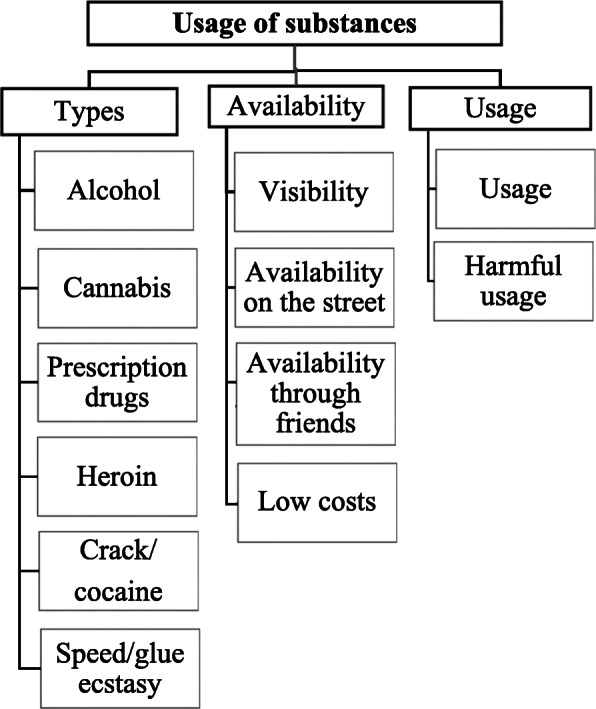
Perceptions about usage of substances among male Syrian refugees in Germany

In the FGDs, various different substances were discussed in the FGDs including alcohol, marihuana and prescription drugs*.* (Fig. 1).

It was emphasized that alcohol use was not common in Syria but use of substances is common in refugees in Germany. (Supplemental Material, Fig. 1) (Theme 1, sub-theme one: Types of substances).

*I mean also in Islam, so in the Quran everything that is* [unintelligible] *everything that is* [unintelligible] *harmful to the body is also prohibited, but alcohol is expressly prohibited, then people say yes, then I don't drink alcohol now, but maybe cannabis, for example consuming, smoking, for example rolling a joint, would then also be okay for many, more accept / more acceptable than or more acceptable than, um /.*

Participants believed that substance use behaviors, especially alcohol use, had changed after immigrating to Germany. Changes in behavior were largely attributed to changes in availability of substances. Substance use was portrayed as normalized in Germany, particularly alcohol use. (Theme 1, sub-theme 2: Availability of substances).

*You know, okay, I have now everything behind me, the war, for example the home, for example the home country, everything behind me, also the escape, the different countries on the, on the Balkan route, that is now the best known. Now I'm finally here. And that, yes, and then you are here in a yes, in a foreign country, ….. But, but you can buy drugs anywhere, on every corner.*

In the refugees` narratives, the easy availability contributed to perceptions that the access to substances was unregulated in Germany. Accordingly, in the narratives there was no difference between use and problematic substance use. (Theme 1, subtheme 3: Use of substances)

*Yes, in a foreign country, for example where you can have everything, you can try everything. No matter whether it is legal or illegal.*

### Theme 2: norms and rules

**Fig. 2:**
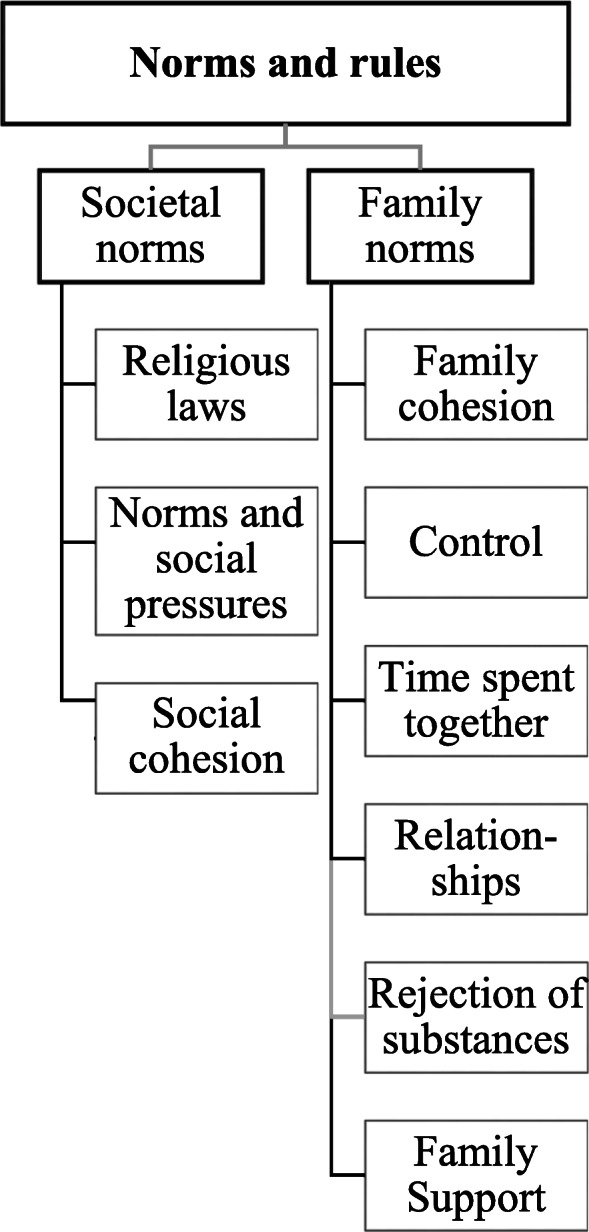
Perceptions about norms and rules among male Syrian refugees in Germany

Societal norms and rules featured particularly strongly in the FGDs. (Fig. 2) Informants described societal and family norms and rules of rejection of substance use.

Additionally, Moslem religion was considered a protective factor (Theme 2, subtheme one: Societal norms).

*With us, society sometimes puts pressure on people who are addicted, for example to alcohol, that is not so acceptable, so there are societies with us that are more conservative and they always make sure that I drink, but the others don't have to get along. ... And we came here young and then there is ... no control, or no one who says, or who sees it, and then you think you can try it.*

Participants reported, however, that since the war in Syria, rules have changed, and substance use became more acknowledged. Use became not only acknowledged but became a way of protesting against the increasingly enforced religious norms imposed by the Islamic State (ISIS).

*ISIS has occupied my city and has forbidden all types of tobacco and hashish.*

Informants perceived that social norms in Germany were different and substance use is tolerated by German society as a whole. Furthermore, openness towards substance use was often seen as a typical norm of Western and especially of the German society.

*And you get real trouble when you smoke, and when you buy or sell ... and suddenly, because it was really forbidden. And then I used hashish at home because it was forbidden. Then you want this relief now, I'm finally here. And that's how it sometimes starts. Either way, I know that is how it is now, so that is how it started with many, many.*

In addition to the societal norms, family norms and support featured heavily in participants’ narratives and seemed to act as a protective force against substance use and the development of addictions.

*I think in Syria we live in families and that makes more control and we had less problems than we were in Syria because there are many, there is always someone to stand behind me and to give me support, and I think if you have a lot of time [in Germany] and also a lot of problems, maybe you are looking for someone who is someone for people who has the drugs to buy or something.*

Additionally, participants expressed that the family was the main reason in preventing substance abuse due to family cohesion, family support and the amount of time spent with the family. (Theme 2, subtheme 3: Family norms)

*I would like, yes if you just, yes, so it is always somehow how it is in the* [unintelligible] *East, and even in Latin countries it is that people are strongly connected in a family. Yes, and there are advantages and disadvantages, depending on who you ask. A positive aspect of this is that it is difficult to go wrong if you are always controlled by the family.*

### Theme 3: influencing factors

**Fig. 3:**
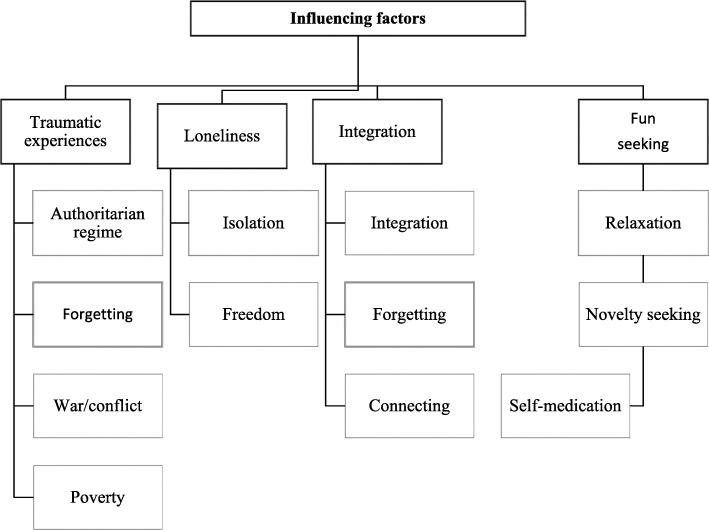
Perceptions about factors influencing substance use among male Syrian refugees in Germany

A theme that cut across ecological levels was the influence of traumatic experiences. (Fig. 3).

Participants reported that many refugees are using substances to escape from memories of past traumatic events in the country of origin (Theme 3, subtheme one: Escaping the past).

*Yes, I mean, so I also think that they, so many, refugees, are now also taking drugs, so of course also because of the war, because of the whole thing so stress and uh yes and yes so I can also confirm that, So many, many now think that this is a solution, they do not know that this is just a temporary or just really provisional, which means provisional. I mean really a short-term solution, maybe for a moment, for a few hours [um] and that does not actually solve a problem, that is actually that is the problem, […]. I'm stressed so often now [umm rough / ]so then I smoke something or consume something, then [uh] it will at least now better for a few hours, but then you also solve no problem [… ] you flee here from the problems, so somehow you try this [… ], I am now pushing this confrontation with my problems away, so that's actually how it is, but at some point so I have to meet them, so uh and find a right solution.*

Additionally, escaping the past was perceived as escaping from the loss of family support. Therefore, one of the main topics was the not only the loss of family support and separation from the family but worries about the family members they had to leave behind.

*I think the side effects of fleeing are not over yet. So far, we have most Syrians, someone has stayed there in Syria and so far we have many problems, they are not safe and we always think of these people. And I think if you want to take drugs then I think the family is very important and that's all for me.*

In addition, another influencing factor for substance use was escaping the present (Theme 3, subtheme 2: Escaping the present). Study participants explained for themselves that substance use among refugees was not solely caused by experiences and memories, but also by the context in the host country related to an expectation–reality gap, leading to the loss of an imagined integration into the host society, which causes distress. In these conditions, loneliness, boredom, and lack of connection was perceived and substance use as a way of escaping these feelings.

*Therefore, escape definitely played a big role. So where people take drugs. So since every new society is a strange society, where you don't even know about it, and then comes language problems, for example, because you can't master the language so quickly. And in this society, so to find yourself again and so. Then drugs is the fastest way.*

In the FDGs, there was a sense of individuals feeling alone and lacking social connections; this could be due to relationship disruptions during resettlement, and trouble connecting with the new community. Additionally, another influencing factor for using substances is to actively manage the situation in the host country and also as a means to achieve goals such as connecting to other people and having fun.

*Where you can quickly find friends who have the same things as him and stuff. Then it feels a little better and so. Good mood. Yes, so it is actually so. Escape plays a big role.*

Substance use is perceived as escaping past and present problems as well as actively seeking ways to manage the present. Therefore, substance use is influenced by actively leaving behind problems.

*So, I think that with the help of addiction, or drugs, you can leave the problems, difficulties, stress in life.*

### Theme 4: barriers of accessing support for substance use

We identified two major factors that impede refugees’ access to psychosocial services: 1) stigma, and shame. (Supplemental Material. Fig. 4) A major barrier was stigma, as underlined by the following statement:

*Generally, that when you say I need psychosocial support or treatment for something and then people say, "Oh he's crazy!" Either way, it's such a shame, so you're ashamed to say, "Yeah, I need psychosocial support or treatment."*

Additionally, it was reported that seeking support for substance use problems is accompanied by shame and a fear that others would have negative attitudes towards seeking professional help.

## Discussion

This study investigated the way in which Syrian refugees understand substance use as well as the perceived causes, risk and protective factors for substance abuse at the individual, family, community, societal and cultural level. Themes identified can be situated within trauma and social context frameworks with substance use as a way of escaping experiences and present living situations. Participants highlighted how substance use was co-produced in their perception by easy availability in Germany (theme 1), societal and family norms (theme 2), and a way of escaping both war related past-experiences, and present distress due to a lack of integration (theme 3). Other important aspects were the fear and shame of negative attitudes of others towards seeking professional help.

Our findings of perceptions of visibility and availability of substances are supported by previous studies [27, 48]. Our study extends these findings by adding knowledge to the group of refugees from Syria living in Germany. Low alcohol use in Syria was perceived as related to religion in Syria (20–22) whereas refugees perceived substance use as widely acceptable and available in German society. Easy availability of substances in the host country was therefore a major topic. The perception of the influence of easy availability is in line with research suggesting that strategies regulating availability (e.g. restrictions on drinking in public, regulations on alcohol advertising) are cost-effective policy options to reduce the harmful use of alcohol [61, 62].

Secondly, our findings that differences in social norms are associated with an increase in substance use has been reported before in a variety of refugees groups, such as refugees from Iraq living in the United States [27]. This perception of alcohol as “taboo” is in line with concepts in predominantly Islamic countries. However, in the FDGs it was reported that behaviors that were previously held as “taboo” have enjoyed new levels of acceptance and tolerance in the last 5–10 years. Therefore, the perceptions of refugees maybe in line with changing habits in using substances in the home country. Major themes in the FDGs are connectedness and family norms. The role of family norms is in line with findings that family support is negatively associated with substance use [63, 64] and substance use by parents is associated with early substance initiation among adolescents [48, 65].

Thirdly, escaping memories of exposure to war - related events was perceived as contributing to substance use. Escaping from the past and the present might be a specific context factors for using substances by refugees. This perception is in line with quantitative studies, which found a significant association between cumulative trauma exposure and harmful alcohol use [66, 67]. An explanatory model for this relationship is the “self-medication theory” [68] or the “tension reduction theory” [69] according to which individuals may engage in substance use to escape and avoid memories related to traumatic experiences. The dysfunctional avoidance model supports these findings [70]. Fourthly, managing the present and connecting to the host country was perceived as another reason for using substances. This finding is in line with research on refugee adolescents [71, 72], suggesting that strong relationships and feelings of belonging contribute to reduced levels of mental distress [73]. Previously, acculturation and assimilation were theoretical models, which suggest that stress of adjusting to life in a new country is related to substance abuse [74]. In our study, substance use was perceived as an active coping behavior to increase acceptance and belonging to the host country.

Finally, we found that religion and cultural values informed ways of seeking help for substance use. The role of religion in affecting substance use will be addressed separately in another paper. This finding is in line with the results of other studies among refugees from Syria, suggesting that their perceptions towards mental illness and receiving help from health professionals are often negative [75, 76]. While some Syrians may seek help from health professionals, some may also be suspicious towards health professionals.

Our study has several strengths*.* We identified the trauma model as one explanatory model: participants were likely to view the causes for substance use as rising from the violence and conflict in the country of origin. Additionally, substance use is not perceived as a medical or psychosocial problem which would need intervention. On the contrary lack of family support is perceived as one reason for substance use. By choosing focus groups as an inquiry method, collective knowledge of a group is unlocked exceeding the sum of singular knowledge, stimulating new ideas in individuals and interviewer while moderation effects are minimized. (25) A further strength of our study was that inclusion of non German – speaking refugees was guaranteed using a professional Syrian interpreter, who assisted in translations during the interview process whenever it was necessary. This also counteracted a possible linguistic and cultural bias. Nevertheless, the study has some limitations. Use of focus groups might have been a limitation, as participants are less likely to disclose very sensitive issues because of social desirability bias, and participants might be less likely to participate in research because of stigma around substance use. However unlike in interviews, the researcher in focus groups takes a peripheral, rather than a centre-stage role. The role of the researcher as facilitator rather than investigator might allow other dynamics of the discussion. Focus groups therefore might facilitate discussion between participants and generate a debate about the collective views of substance use and the meanings that lie behind those views.

It cannot be ruled out that presence of a person from the same culture caused a certain level of inhibition among participants. Moreover, it might be especially difficult to talk about practices which are not culturally acceptable such as alcohol and drug use. Since the study utilised focus group methodology rather than individual interviews and included participants knowledgeable about the challenges facing Syrian refugees, the results only capture the perspectives of others on individual difficulties rather than fully capturing the direct experience of individuals. This was due to not specifying those experiencing substance use difficulties themselves. In addition, a female facilitator with the help of a male interpreter conducted the FDGs. While the authors made the judgment that this would be appropriate in this instance, there is the possibility that the gender of the facilitators may have influenced the results such that participants were less comfortable to disclose their own experiences. Additionally, due to the qualitative nature of the study the results cannot be generalized to the entire community of Syrian refugees nor to the highly heterogeneous population of all refugees. Our results require replication in larger samples. We focused on male refugees as more knowledge of male refugees is critical but future studies could additionally include women. Furthermore, a limitation might be the design of focus group design. There are inherent possibilities for bias and subjectivity in qualitative research. However, during analyses we were aware that researchers’ prior experiences, interests, beliefs, culture, and expectations might influence analysis. Thus the facilitator, the interpreter, and the raters discussed issues of reflexivity throughout analysis.

The analyses of the FGD have been used to tailor interventions for refugees and will be used to develop a specific assessment instrument. The results suggest the need for further research in the are of substance use among refugees including research into barriers for seeking help.

## Conclusion

To our knowledge, this is the first study to explore Syrian refugees’ perceptions of substance use in Germany. Findings provide support for the trauma-focused and psychosocial models. We realized that Syrian refugees explanatory model of substance use is rooted in religious, social, family, and individual contexts and past and present experiences. Our findings might provide helpful insights for tailoring interventions to the needs of refugees with substance use problems. Firstly, the potential influence of cultural values on seeking help suggests that special attention should be paid to offer transparent and reassuring information about services. Secondly, assessment instruments used in routine practice should be reviewed to make sure that they appropriately cover all culture specific aspects related to problematic use. Finally, the subjective influence of trauma and its consequences on substance use highlights the importance of integrating specific interventions related to these problems in substance abuse services for refugee populations. Results obtained in the focus groups had practical implications: they have been used to adapt an intervention to reduce substance use and inform the development of a culture by being a refugee sensitive assessment instrument.

From a public health perspective, our results highlight the need to respond to substance use with a multi-sectoral social and psychiatric approach that pays attention to experiences (e.g., war experiences), present living conditions (e.g., living alone) and notions of culturally accepted behavior (e.g., help seeking) in an ecological model [42]. Although previous studies have investigated such a model for mental health among refugees [77, 78], this paper adds specific knowledge towards the perceptions of substance use by refugees. In clinical practice, social factors such as traumatic experiences and perceived context in the host country should therefore guide concepts to support refugees with substance use.

## Supplementary Information

**Additional file 1.**

## Data Availability

The datasets analysed during this study are available from the corresponding author on reasonable request.
